# 1-(4-Bromo­phen­yl)-3-(3-chloro­propan­oyl)thio­urea

**DOI:** 10.1107/S1600536814011209

**Published:** 2014-05-21

**Authors:** Hamza M. Abosadiya, Siti Aishah Hasbullah, Bohari M. Yamin, Adibatul H. Fadzil

**Affiliations:** a School of Chemical Sciences and Food Technology, Universiti Kebangsaan Malaysia, 43600 Bangi, Selangor D.E. , Malaysia; b Low Carbon Research Group, School of Chemical Sciences and Food Technology, Universiti Kebangsaan Malaysia, 43600 Bangi, Selangor D.E. , Malaysia; c Faculty of Applied Sciences, Universiti Teknologi MARA (UiTM), 40450 Shah Alam, Selangor D.E. , Malaysia

## Abstract

The title compound, C_10_H_10_BrClN_2_OS, adopts a *trans*–*cis* conformation with respect to the position of the 3-chloro­propanoyl and 4-bromo­phenyl groups, respectively, against the thiono C=S bond across their C—N bonds. The benzene ring makes a dihedral angle of 9.55 (16)° with the N_2_CS thio­urea moiety. Intra­molecular N—H⋯O and C—H⋯S hydrogen bonds occur. In the crystal, mol­ecules are linked into chains along the *c-*axis direction by N—H⋯S, C—H⋯S and C—H⋯O hydrogen bonds.

## Related literature   

For the crystal structures of related compounds, see: Othman *et al.* (2010 ▶). For bond-length data, see: Allen *et al.* (1987 ▶).
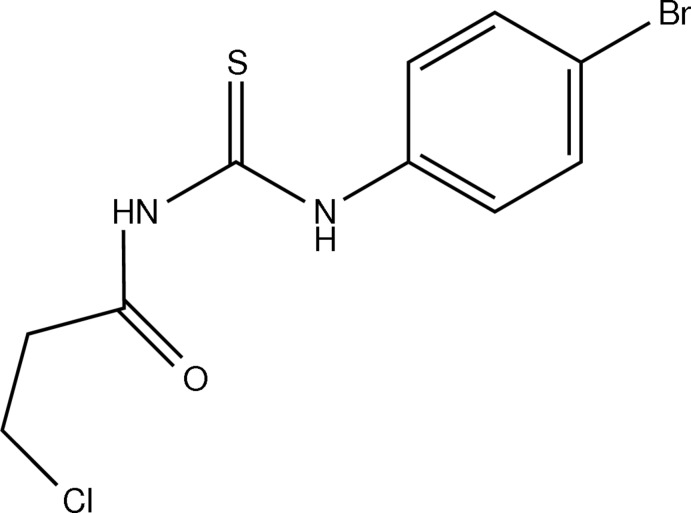


## Experimental   

### 

#### Crystal data  

C_10_H_10_BrClN_2_OS
*M* *_r_* = 321.62
Triclinic, 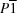

*a* = 5.3899 (4) Å
*b* = 8.3705 (5) Å
*c* = 13.7369 (8) Å
α = 91.209 (2)°
β = 96.417 (2)°
γ = 92.731 (2)°
*V* = 614.96 (7) Å^3^
*Z* = 2
Mo *K*α radiation
μ = 3.71 mm^−1^
*T* = 296 K
0.46 × 0.45 × 0.15 mm

#### Data collection  

Bruker SMART APEX CCD diffractometer
Absorption correction: multi-scan (*SADABS*; Bruker, 2009 ▶) *T*_min_ = 0.280, *T*_max_ = 0.606
11958 measured reflections
2405 independent reflections
2053 reflections with *I* > 2σ(*I*)
*R*_int_ = 0.129

#### Refinement  

*R*[*F* ^2^ > 2σ(*F* ^2^)] = 0.043
*wR*(*F* ^2^) = 0.111
*S* = 1.11
2405 reflections
154 parameters
2 restraints
H atoms treated by a mixture of independent and constrained refinement
Δρ_max_ = 0.44 e Å^−3^
Δρ_min_ = −0.67 e Å^−3^

### 

Data collection: *SMART* (Bruker, 2009 ▶); cell refinement: *SAINT* (Bruker, 2009 ▶); data reduction: *SAINT*; program(s) used to solve structure: *SHELXS97* (Sheldrick, 2008 ▶); program(s) used to refine structure: *SHELXL97* (Sheldrick, 2008 ▶); molecular graphics: *SHELXTL* (Sheldrick, 2008 ▶); software used to prepare material for publication: *SHELXTL* and *PLATON* (Spek, 2009 ▶).

## Supplementary Material

Crystal structure: contains datablock(s) global, I. DOI: 10.1107/S1600536814011209/rk2427sup1.cif

Structure factors: contains datablock(s) I. DOI: 10.1107/S1600536814011209/rk2427Isup2.hkl

Supporting information file. DOI: 10.1107/S1600536814011209/rk2427Isup3.cml

CCDC reference: 1003286

Additional supporting information:  crystallographic information; 3D view; checkCIF report

## Figures and Tables

**Table 1:** Hydrogen-bond geometry (Å, °)

*D*—H⋯*A*	*D*—H	H⋯*A*	*D*⋯*A*	*D*—H⋯*A*
N2—H2⋯O1	0.87 (3)	1.90 (3)	2.623 (4)	140 (4)
C6—H6⋯S1	0.93	2.56	3.222 (4)	128
N1—H1⋯S1^i^	0.87 (2)	2.53 (2)	3.376 (3)	166 (4)
C2—H2*B*⋯S1^i^	0.97	2.79	3.707 (4)	157
C9—H9⋯O1^ii^	0.93	2.52	3.444 (5)	172

Symmetry codes: (i) 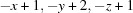; (ii) 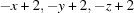.

**Fig. 1.**
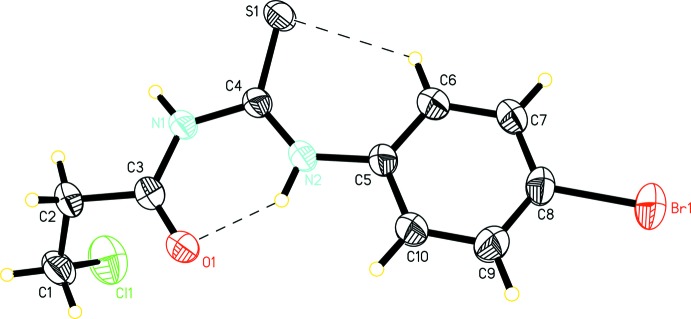
The molecular structure of title compound with the atom numbering scheme. The displacement ellipsoids are drawn at 50% probability level. The H atoms are presented as a small spheres of arbitrary radius. The dashed lines indicate intramolecular hydrogen bonds.

**Fig. 2.**
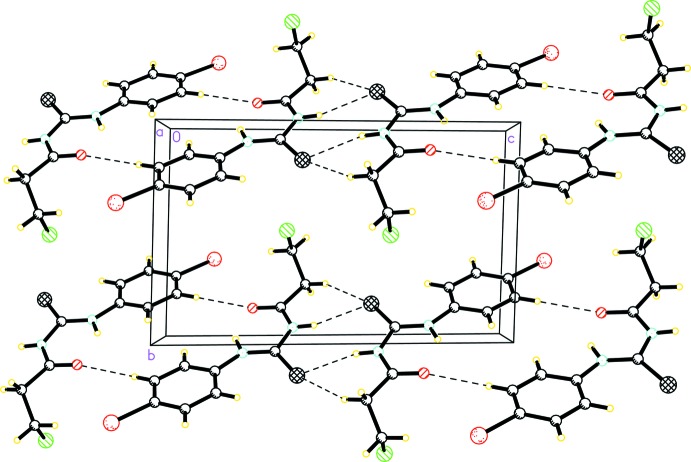
The crystal packing of the title compound viewed down a axis. The dashes lines indicate hydrogen bonds.

## supplementary crystallographic information

### 1. Comment

The title compound is analogous to the previously reported *N*-(3-chloropropanoyl)-*N*'-phenylthiourea (Othman *et al.*, 2010) except the bromine atom is at position-4 of the phenyl ring (Fig. 1). The molecule has *trans*-*cis* configuration with respect to the position of the 3-chloropropanoyl and 4-bromophenyl groups, respectively, against the thiono C═S bond across their C4–N1 and C4–N2 bonds. The whole molecule is not planar. The (S1/N1/N2/C2/C3/C4) thiourea moiety and the benzene ring (C5-C10) are planar with maximum deviation of 0.036 (4) Å for C3 atom from the least square plane of the thiourea moiety. The benzene ring makes dihedral angle with the thiourea moiety of 9.55 (16)°, very big reduction compared to the analog, *N*-(3-chloropropanoyl)-*N*'-phenylthiourea of 82.62 (10)°. The bond lengths and angles are in normal ranges (Allen *et al.*, 1987). There are N2–H2···O1 and C6–H6···S1 intramolecular hydrogen bonds. In the crystal packing, the molecules are linked by N1–H1···S1^i^, C2–H2B···S1^i^ and C9–H9···O1^ii^ intermolecular hydrogen bonds form one-dimensional chains along the *c* axis (Fig. 2). Symmetry codes: (i) -*x*+1, -*y*+2, -*z*+1; (ii) -*x*+2, -*y*+2, -*z*+2.

### 2. Experimental

An acetone solution (30 mL) of 4-bromoaniline (0.01 mol, 1.72 g) was added dropwise into a two-necked round-bottomed flask containing 3-chloropropanoylisothiocyanate (0.01 mol). The mixture was refluxed for about 4 h, filtered into a beaker and left to evaporate at room temperature. The filtrate gave colourless crystals after 5 days on slow evaporation of the solvent (yield 79%).

### 3. Refinement

The C based H atoms were positioned geometrically with C–H = 0.93-0.97 Å and constrained to ride on their parent atoms with *U*_iso_(H) = 1.2*U*_eq_(C). The amino H atoms ware located in difference Fourier map and refined freely with using SHELXL instruction 'DFIX 0.87 0.01'.

### Crystal data

**Table:**

C_10_H_10_BrClN_2_OS	*Z* = 2
*M**_r_* = 321.62	*F*(000) = 320
Triclinic, *P*1	*D*_x_ = 1.737 Mg m^−^^3^
Hall symbol: -P 1	Mo *K*α radiation, λ = 0.71073 Å
*a* = 5.3899 (4) Å	Cell parameters from 7910 reflections
*b* = 8.3705 (5) Å	θ = 2.9–25.9°
*c* = 13.7369 (8) Å	µ = 3.71 mm^−^^1^
α = 91.209 (2)°	*T* = 296 K
β = 96.417 (2)°	Block, colourless
γ = 92.731 (2)°	0.46 × 0.45 × 0.15 mm
*V* = 614.96 (7) Å^3^	

### Data collection

**Table:**

Bruker SMART APEX CCD diffractometer	2405 independent reflections
Radiation source: fine-focus sealed tube	2053 reflections with *I* > 2σ(*I*)
Graphite monochromator	*R*_int_ = 0.129
Detector resolution: 83.66 pixels mm^-1^	θ_max_ = 26.0°, θ_min_ = 3.0°
ω scans	*h* = −6→6
Absorption correction: multi-scan (*SADABS*; Bruker, 2009)	*k* = −10→10
*T*_min_ = 0.280, *T*_max_ = 0.606	*l* = −16→16
11958 measured reflections	

### Refinement

**Table:**

Refinement on *F*^2^	Secondary atom site location: difference Fourier map
Least-squares matrix: full	Hydrogen site location: inferred from neighbouring sites
*R*[*F*^2^ > 2σ(*F*^2^)] = 0.043	H atoms treated by a mixture of independent and constrained refinement
*wR*(*F*^2^) = 0.111	*w* = 1/[σ^2^(*F*_o_^2^) + (0.0381*P*)^2^ + 0.5365*P*] where *P* = (*F*_o_^2^ + 2*F*_c_^2^)/3
*S* = 1.11	(Δ/σ)_max_ = 0.001
2405 reflections	Δρ_max_ = 0.44 e Å^−^^3^
154 parameters	Δρ_min_ = −0.67 e Å^−^^3^
2 restraints	Extinction correction: *SHELXL97* (Sheldrick, 2008), Fc^*^=kFc[1+0.001xFc^2^λ^3^/sin(2θ)]^-1/4^
Primary atom site location: structure-invariant direct methods	Extinction coefficient: 0.067 (5)

### Special details

**Table:**

Geometry. All s.u.'s (except the s.u. in the dihedral angle between two l.s. planes) are estimated using the full covariance matrix. The cell s.u.'s are taken into account individually in the estimation of s.u.'s in distances, angles and torsion angles; correlations between s.u.'s in cell parameters are only used when they are defined by crystal symmetry. An approximate (isotropic) treatment of cell s.u.'s is used for estimating s.u.'s involving l.s. planes.
Refinement. Refinement of *F*^2^ against ALL reflections. The weighted *R*-factor *wR* and goodness of fit *S* are based on *F*^2^, conventional *R*-factors *R* are based on *F*, with *F* set to zero for negative *F*^2^. The threshold expression of *F*^2^ > σ(*F*^2^) is used only for calculating *R*-factors(gt) *etc*. and is not relevant to the choice of reflections for refinement. *R*-factors based on *F*^2^ are statistically about twice as large as those based on *F*, and *R*-factors based on ALL data will be even larger.

### Fractional atomic coordinates and isotropic or equivalent isotropic displacement parameters (Å^2^)

**Table:**

	*x*	*y*	*z*	*U*_iso_*/*U*_eq_	
Br1	0.28574 (8)	0.63531 (5)	1.11594 (3)	0.0559 (2)	
Cl1	0.8887 (2)	1.50961 (14)	0.64873 (11)	0.0707 (4)	
S1	0.40834 (18)	0.83621 (11)	0.60404 (6)	0.0447 (3)	
O1	1.0855 (5)	1.1309 (3)	0.74807 (18)	0.0481 (7)	
N1	0.7900 (5)	1.0463 (3)	0.6239 (2)	0.0348 (6)	
N2	0.7069 (6)	0.9295 (4)	0.7667 (2)	0.0387 (7)	
C1	1.1700 (7)	1.4130 (4)	0.6359 (3)	0.0457 (9)	
H1A	1.2690	1.4749	0.5939	0.055*	
H1B	1.2667	1.4073	0.6996	0.055*	
C2	1.1149 (7)	1.2461 (4)	0.5923 (3)	0.0429 (9)	
H2A	1.2689	1.2024	0.5759	0.051*	
H2B	1.0022	1.2509	0.5324	0.051*	
C3	0.9979 (7)	1.1373 (4)	0.6630 (3)	0.0371 (8)	
C4	0.6423 (6)	0.9385 (4)	0.6716 (2)	0.0313 (7)	
C5	0.5977 (6)	0.8495 (4)	0.8422 (2)	0.0332 (7)	
C6	0.3732 (7)	0.7605 (5)	0.8312 (3)	0.0450 (9)	
H6	0.2838	0.7447	0.7695	0.054*	
C7	0.2820 (7)	0.6947 (5)	0.9131 (3)	0.0452 (9)	
H7	0.1311	0.6347	0.9066	0.054*	
C8	0.4156 (7)	0.7187 (4)	1.0035 (3)	0.0382 (8)	
C9	0.6388 (7)	0.8042 (5)	1.0150 (3)	0.0485 (9)	
H9	0.7287	0.8193	1.0766	0.058*	
C10	0.7283 (7)	0.8680 (5)	0.9334 (3)	0.0481 (10)	
H10	0.8818	0.9251	0.9405	0.058*	
H1	0.742 (7)	1.058 (5)	0.5620 (10)	0.041 (10)*	
H2	0.835 (5)	0.990 (4)	0.791 (3)	0.057 (13)*	

### Atomic displacement parameters (Å^2^)

**Table:**

	*U*^11^	*U*^22^	*U*^33^	*U*^12^	*U*^13^	*U*^23^
Br1	0.0605 (3)	0.0655 (3)	0.0444 (3)	−0.0072 (2)	0.0209 (2)	0.0123 (2)
Cl1	0.0667 (7)	0.0585 (7)	0.0908 (9)	0.0081 (6)	0.0226 (6)	0.0126 (6)
S1	0.0513 (6)	0.0445 (5)	0.0338 (5)	−0.0216 (4)	−0.0061 (4)	0.0080 (4)
O1	0.0475 (15)	0.0584 (16)	0.0349 (14)	−0.0235 (13)	−0.0007 (11)	0.0039 (12)
N1	0.0375 (15)	0.0362 (14)	0.0288 (14)	−0.0128 (12)	0.0007 (12)	0.0049 (12)
N2	0.0387 (16)	0.0433 (16)	0.0315 (15)	−0.0178 (13)	0.0003 (12)	0.0068 (12)
C1	0.043 (2)	0.0406 (19)	0.052 (2)	−0.0126 (16)	0.0040 (17)	0.0059 (17)
C2	0.041 (2)	0.046 (2)	0.041 (2)	−0.0149 (16)	0.0104 (16)	0.0050 (16)
C3	0.0387 (19)	0.0356 (17)	0.0367 (19)	−0.0070 (14)	0.0072 (15)	0.0016 (14)
C4	0.0354 (17)	0.0270 (15)	0.0308 (16)	−0.0041 (13)	0.0025 (13)	0.0032 (12)
C5	0.0355 (17)	0.0333 (16)	0.0303 (16)	−0.0046 (14)	0.0031 (13)	0.0057 (13)
C6	0.045 (2)	0.051 (2)	0.0366 (19)	−0.0174 (17)	0.0007 (15)	0.0018 (16)
C7	0.042 (2)	0.050 (2)	0.042 (2)	−0.0175 (17)	0.0049 (16)	0.0050 (17)
C8	0.0416 (19)	0.0400 (18)	0.0353 (18)	0.0004 (15)	0.0137 (15)	0.0062 (14)
C9	0.046 (2)	0.065 (2)	0.0323 (18)	−0.0089 (19)	−0.0012 (16)	0.0070 (17)
C10	0.039 (2)	0.066 (2)	0.036 (2)	−0.0209 (18)	−0.0008 (15)	0.0054 (18)

### Geometric parameters (Å, º)

**Table:**

Br1—C8	1.898 (3)	C2—C3	1.514 (4)
Cl1—C1	1.776 (4)	C2—H2A	0.9700
S1—C4	1.669 (3)	C2—H2B	0.9700
O1—C3	1.213 (4)	C5—C10	1.369 (5)
N1—C3	1.375 (4)	C5—C6	1.382 (5)
N1—C4	1.397 (4)	C6—C7	1.391 (5)
N1—H1	0.869 (10)	C6—H6	0.9300
N2—C4	1.319 (4)	C7—C8	1.370 (5)
N2—C5	1.414 (4)	C7—H7	0.9300
N2—H2	0.864 (10)	C8—C9	1.362 (5)
C1—C2	1.511 (5)	C9—C10	1.376 (5)
C1—H1A	0.9700	C9—H9	0.9300
C1—H1B	0.9700	C10—H10	0.9300
			
C3—N1—C4	128.1 (3)	N2—C4—N1	114.9 (3)
C3—N1—H1	117 (2)	N2—C4—S1	127.4 (2)
C4—N1—H1	115 (2)	N1—C4—S1	117.7 (2)
C4—N2—C5	133.1 (3)	C10—C5—C6	119.1 (3)
C4—N2—H2	116 (3)	C10—C5—N2	115.2 (3)
C5—N2—H2	111 (3)	C6—C5—N2	125.7 (3)
C2—C1—Cl1	110.8 (3)	C5—C6—C7	119.4 (3)
C2—C1—H1A	109.5	C5—C6—H6	120.3
Cl1—C1—H1A	109.5	C7—C6—H6	120.3
C2—C1—H1B	109.5	C8—C7—C6	119.7 (3)
Cl1—C1—H1B	109.5	C8—C7—H7	120.1
H1A—C1—H1B	108.1	C6—C7—H7	120.1
C1—C2—C3	111.3 (3)	C9—C8—C7	121.3 (3)
C1—C2—H2A	109.4	C9—C8—Br1	118.9 (3)
C3—C2—H2A	109.4	C7—C8—Br1	119.7 (3)
C1—C2—H2B	109.4	C8—C9—C10	118.6 (4)
C3—C2—H2B	109.4	C8—C9—H9	120.7
H2A—C2—H2B	108.0	C10—C9—H9	120.7
O1—C3—N1	123.0 (3)	C5—C10—C9	121.8 (3)
O1—C3—C2	121.5 (3)	C5—C10—H10	119.1
N1—C3—C2	115.5 (3)	C9—C10—H10	119.1
			
Cl1—C1—C2—C3	−68.4 (4)	C10—C5—C6—C7	1.4 (6)
C4—N1—C3—O1	2.0 (6)	N2—C5—C6—C7	−176.8 (4)
C4—N1—C3—C2	−178.8 (3)	C5—C6—C7—C8	−0.1 (6)
C1—C2—C3—O1	−47.9 (5)	C6—C7—C8—C9	−0.9 (6)
C1—C2—C3—N1	132.9 (3)	C6—C7—C8—Br1	177.8 (3)
C5—N2—C4—N1	173.3 (4)	C7—C8—C9—C10	0.4 (6)
C5—N2—C4—S1	−6.9 (6)	Br1—C8—C9—C10	−178.3 (3)
C3—N1—C4—N2	3.1 (5)	C6—C5—C10—C9	−1.9 (7)
C3—N1—C4—S1	−176.7 (3)	N2—C5—C10—C9	176.5 (4)
C4—N2—C5—C10	178.7 (4)	C8—C9—C10—C5	1.0 (7)
C4—N2—C5—C6	−3.0 (7)		

### Hydrogen-bond geometry (Å, º)

**Table:**

*D*—H···*A*	*D*—H	H···*A*	*D*···*A*	*D*—H···*A*
N2—H2···O1	0.87 (3)	1.90 (3)	2.623 (4)	140 (4)
C6—H6···S1	0.93	2.56	3.222 (4)	128
N1—H1···S1^i^	0.87 (2)	2.53 (2)	3.376 (3)	166 (4)
C2—H2*B*···S1^i^	0.97	2.79	3.707 (4)	157
C9—H9···O1^ii^	0.93	2.52	3.444 (5)	172

Symmetry codes: (i) −*x*+1, −*y*+2, −*z*+1; (ii) −*x*+2, −*y*+2, −*z*+2.
